# Group-based versus one-to-one occupational therapy to improve ADL ability in people with chronic conditions: a pilot and feasibility RCT of the Go:OT study

**DOI:** 10.1186/s40814-026-01855-1

**Published:** 2026-06-06

**Authors:** Cecilie von Bülow, Signe B Bojesen, Vita Hagelskjær, Kristina T Nielsen, Eva E Wæhrens

**Affiliations:** 1 Occupation-centered Occupational Therapy, The Parker Institute, Bispebjerg and Frederiksberg Hospital, University of Copenhagen, Copenhagen, Denmark; 2 Rehabilitation Centre, Rødovre Municipality, Rødovre, Denmark; 3 Occupational Science, User Perspectives and Community-based Interventions, Department of Public Health, University of Southern Denmark, Odense, Denmark; 4 Research Centre for Care and Rehabilitation, VIA University College, Aarhus, Denmark; 5 Department of Occupational Therapy, University College of Northern Denmark, Aalborg, Denmark

**Keywords:** ADL ability, Complex interventions, Occupational Therapy Intervention Process Model (OTIPM), Rehabilitation, AMPS, ADAPT program

## Abstract

**Background:**

Occupational therapy interventions can improve the ability to perform activities of daily living (ADL) in individuals with chronic conditions. The group-based ADAPT program was developed as an alternative to usual one-to-one occupational therapy (UOT), aiming to enhance ADL ability through structured problem-solving processes and implementation of adaptational strategies. This pilot and feasibility study evaluated the feasibility of delivering ADAPT 3.0 in a municipal setting and informed the design of a future full-scale randomised controlled trial (RCT).

**Methods:**

A two-armed pilot RCT was conducted in a Danish municipality, comparing outcomes of ADAPT 3.0 to UOT. Eligible clients (≥ 18 years, chronic conditions, decreased ADL ability) were randomised to either intervention or control group. Outcomes included recruitment and retention, trial participation, impact on staff, access to UOT documentation, completion of outcome measures, and fidelity to the ADAPT manual. Descriptive statistics, logbook notes, and predefined progression criteria guided the evaluation.

**Results:**

Twelve clients and four ADAPT-trained occupational therapists participated. ADAPT 3.0 was delivered with high fidelity and dose. Most progression criteria were met, including access to UOT documentation and successful delivery of ADAPT 3.0. However, recruitment was slower than anticipated, and clients in the UOT group reported to receive limited trial-related information. Completion rates of follow-up evaluations were acceptable in the ADAPT group but lower in the UOT group. Minor structural and procedural challenges were identified.

**Conclusion:**

The study supports the feasibility of delivering ADAPT 3.0 in a municipal setting and progressing to a full-scale RCT. To ensure robust trial conduct, key refinements are needed in recruitment procedures, participant information for the UOT group, and clarity of outcome measures.

**Trial registration:**

ClinicalTrials.gov Identifier: NCT05775653.

## Key messages regarding feasibility

- What uncertainties existed regarding the feasibility?
  This study explored uncertainties related to trial design, participant engagement, and intervention delivery in preparation for a full-scale randomised controlled trial (RCT) of ADAPT 3.0. Key focus areas included recruitment and randomisation procedures, participants’ understanding of trial involvement, the impact on occupational therapists delivering ADAPT, access to data on usual occupational therapy (UOT), completion of measures, and fidelity to the ADAPT manual.
- What are the key feasibility findings?
  ADAPT 3.0 was delivered with high fidelity. Most trial procedures were feasible, including access to UOT documentation and delivery of the ADAPT sessions in a municipality setting. Challenges were identified, including slow recruitment, limited participant information in the UOT group, and lack of clarity in certain questionnaire items. Some occupational therapists experienced difficulties with conducting standardised ADL assessments and goal setting, particularly in the UOT group.
- What are the implications of the feasibility findings for the design of the main study?
  The findings support progression to a full-scale trial, provided that recruitment procedures are strengthened (e.g. through pre-screening and shared coordination roles), clearer trial information for the UOT group, minor structural adjustments to ADAPT 3.0, and refinement of outcome measures.

## Introduction

Chronic conditions encompass a wide range of conditions, including arthritis, chronic respiratory conditions, cardiovascular conditions, and diabetes [1]. They can be defined as ‘conditions that last a year or more and require ongoing medical attention and/or limit activities of daily living’ (ADL) [1]. People living with chronic conditions often report increased use of time and physical effort/fatigue when performing ADL tasks [2, 3] including self-care and household chores [4]. Decreased ADL ability may hinder people’s engagement in valued tasks, compromise autonomy, and is associated with diminished quality of life [5–7], increased risk of further functional decline [8–11], hospitalisation, and need for home help [12]. Hence, there is a need for interventions that prevent or mitigate ADL task performance problems and their associated consequences [13].

Occupational therapy (OT) interventions have demonstrated effectiveness in improving ADL ability in people with chronic conditions, primarily through adaptation, i.e. teaching and practicing problem-solving strategies and implementing environmental modifications to improve task performance [13–18]. In Denmark, such OT services are typically delivered as individualised, one-to-one interventions within municipal settings.

The group-based ADAPT program [19–24] was originally developed as an alternative to one-to-one OT. ADAPT aims to improve ADL ability by providing clients with knowledge and skills enabling them to overcome task performance problems. Following the British Medical Research Council’s (MRC) guidance [25] and framework [26] on how to develop and evaluate complex interventions, the first 16-week version of the ADAPT program (ADAPT 1.0) [20] was piloted in a research hospital setting involving women diagnosed with fibromyalgia between 2012 and 2015 [22]. The study included both process and long-term outcomes evaluations [23, 24], which provided preliminary evidence of improved ADL motor ability (i.e. decreased physical effort/fatigue during ADL task performance) [22], enhanced problem-solving skills, mental well-being, and acceptance [24]. Findings also indicated that reducing the number of sessions and adding booster sessions could improve attendance and outcomes and support sustainable gains. Hence, ADAPT 2.0 was revised to include 10 weekly group sessions and two booster sessions (sessions offered post ADAPT program to reinforce key strategies, support ongoing development, and address emerging needs) at weeks 16 and 24.

The content and delivery of ADAPT 2.0 was evaluated for its feasibility when delivered by trained occupational therapists working in real-life municipal settings [21, 27] to groups of clients with various chronic conditions. Across diagnoses, sexes, and age groups, clients consistently found ADAPT to be highly acceptable, while the occupational therapists found it both feasible and meaningful [21, 27], although some deviations from the programme manual were reported, indicating fidelity challenges. Additionally, recruitment difficulties and insufficient participant information were reported [27], highlighting the need for improved fidelity and recruitment procedures. i.e. insights that informed the development of ADAPT 3.0.

In a co-creational process involving OT researchers and municipal stakeholders, ADAPT 2.0 was updated to ADAPT 3.0, integrating contemporary OT theory, models [28], and research evidence [29–31]. A key enhancement was the inclusion of one-to-one ADL evaluations in the clients’ homes (sessions 1 and 10), supporting a more tailored and client-centred intervention [28]. Sessions 2 to 9 remained group-based, as did the two booster sessions (sessions 11 to 12).

To prepare for a full-scale randomised controlled trial (RCT), comparing ADAPT 3.0 to usual occupational therapy (UOT), preliminary insights into UOT were gathered via an internal audit on 27 client-records. UOT was typically delivered one-to-one in the clients’ homes, with content and dose tailored to individual needs based on the occupational therapist’s professional judgement. Clients received a mean of seven sessions (range, 2 to 27), each lasting a mean of 60 min (range, 30 to 90 min). UOT typically included ADL evaluations (standardised or non-standardised), counselling, practicing use of adaptive strategies, performing tasks to restore body functions or physical exercises. These findings confirmed UOT as a relevant comparator for ADAPT in a future RCT.

Prior to the RCT, there was a need to evaluate fidelity to ADAPT 3.0 and address previously identified challenges in trial conduct and design [27]. This pilot and feasibility study was therefore conducted to assess these aspects and resolve remaining uncertainties.

## Methods

### Aims and objectives

In accordance with the framework developed by O’Cathain et al. [32], the aim of this pilot and feasibility study was to evaluate aspects related to (1) trial design, conduct, and processes (recruitment, trial participation, impact of trial on staff, adaptation of trial conduct to local context); (2) measures (completion of measures); and (3) intervention content and delivery (fidelity and dose) of the ADAPT 3.0 program. More specifically, the study objectives were to evaluate:

1. How recruitment procedures worked and if clients accepted randomisation (recruitment and randomisation)
2. If clients felt adequately informed about the study, including its purpose and the extent of evaluations and questionnaires (trial participation)
3. If trial participation had a significant negative impact on the occupational therapists delivering the ADAPT program (impact of trial on staff)
4. If occupational therapists perceived organisational or practical factors to significantly hinder ADAPT program delivery (adaptation of trial conduct to local context)
5. If data informing about UOT could be extracted from client records (adaptation of trial conduct to local context)
6. If outcome evaluations and registration forms were completed (completion of measures).
7. The extent to which the occupational therapists adhered to the ADAPT program manual (fidelity and dose).

Table 1 summarises the objectives and methods applied in this pilot and feasibility study. Based on the aims and objectives, addressing uncertainties related to recruitment, intervention delivery, trial procedures, and contextual fit, as well as the need to evaluate the feasibility of novel aspects of the ADAPT program, this study is designed as an external pilot, conducted prior to launching the full-scale Go:OT (Group versus one-to-one Occupational Therapy) RCT. This decision is supported by the extended conceptual framework for pilot and feasibility studies proposed by Bond et al. [33].

**Table 1:** Domains by O’Cathain [32] with objectives, data collection, analysis methods, and progression criteria

O’Cathain categories	Trial design, conduct, and processes					Measures	Intervention content and delivery
**O’Cathain sub-categories**	**Recruit-ment**	**Trial participation**	**Impact of trial on staff**	**Adaptation of trial conduct to local context**		**Completion of measures**	**Fidelity and dose**
**Objectives**	How recruitment procedures worked and if clients accepted randomisation	If clients felt adequately informed about the study, including its purpose and the extent of evaluations and questionnaires	If trial participation had a significant negative impact on the occupational therapists delivering ADAPT	If occupational therapists perceived organisational/practical factors to significantly hinder adapt programme delivery	If data informing about UOT could be extracted from client records	If outcome evaluations and registration forms were completed	If occupational therapists adhered to the ADAPT program manual
**Data collection methods**	Records to monitor a recruitment rate of four clients per week. Logbook notes	2 questions in the 12-week questionnaire	Registration forms filled out by occupational therapists after each ADAPT session. Logbook notes	Registration forms filled out by ADAPT OTs after each ADAPT session. Logbook notes	Data extraction in the UOT group from client records using prespecified variables. Logbook notes	Outcome evaluations conducted and ADAPT-registration forms filled out by clients and occupational therapists at 12 and 26 weeks. Logbook notes	ADAPT registration forms filled out by the occupational therapists after each ADAPT session
**Data analysis**	Number of clients recruited per week. Logbook summary	Calculate the number of clients feeling inadequate, somewhat adequate, or adequately informed including written elaborations	Calculate the number of 2-point ratings reflecting significant negative side effects and report written elaborations. Logbook summary	Calculate the number of 2-point ratings reflecting significant negative organisational or practical aspects and report written elaborations. Logbook summary	Describe the extent of access to documented information on UOT intervention, in numbers and percentages. Logbook summary	Numbers (%) of outcome evaluations and registration forms completed. Written elaboration informing about reasons for incomplete data. Logbook summary	Describe numbers (%) of session specific key components and sessions delivered including possibly written elaborations informing programme adaptation or deviation
**Progression criteria**	> 12 clients accepting participation and group allocation within a 4-week period	> 80% feeling adequately informed	0% negative side effects of significant importance	0% negative organisational or practical aspects of significant importance	> 80% of the client cases have documented access to information on UOT	> 80% of the outcome evaluations and registration forms are completed	> 80% of the session specific components have been delivered > 80% of the sessions have been delivered

### Study design and organisation

The study was designed as a two-armed parallel-groups RCT with allocation to either ADAPT or UOT. The design of the study adhered to the CONSORT 2010 statement with extension to randomised pilot and feasibility trials [34] and was approved by the Knowledge Centre for Data Registration, in the Capital Region of Denmark (registration number: P-2022-766). 

Primary, secondary, and exploratory outcomes were evaluated at baseline, 12, and 26 weeks from baseline. Data representing perspectives on aspects of trial design and programme delivery among clients having received, and occupational therapists having delivered the ADAPT program were gathered during and immediately after programme delivery.

The study was conducted in collaboration between a research institute and a municipal rehabilitation centre. A local occupational therapist, also employed as a part-time development occupational therapist, served as study coordinator (hereafter referred to as the study coordinator), responsible for screening participants for trial inclusion, performing baseline evaluations, randomising and allocating participants, and coordinating their flow through the trial. Local rehabilitation professionals were also informed about screening criteria to support recruitment.

### Settings

The trial was scheduled to be conducted from March to September 2023 in a Danish municipality. Group-based sessions were held at a municipal rehabilitation centre equipped with a kitchen, bedroom, and living room simulation areas, household equipment (e.g. vacuum cleaner, mop, kitchen services, bed linens, foods, coffee machine), assistive devices, and orthoses. One-to-one sessions (ADAPT sessions 1 and 10) and outcome evaluations were conducted in the clients’ homes. As UOT was not delivered according to a predefined protocol with respect to content, dose, or setting, the description of the UOT setting reflects occupational therapists’ usual clinical practice. Assessments and interventions were primarily conducted in clients’ homes or local environments, and less frequently at the municipal rehabilitation centre or via telephone.

### Participants and recruitment

Potential participants were identified either by the study coordinator based on referral information and brief clinical judgement, or by local rehabilitation professionals during routine clinical contacts.

This initial screening was intended as a pragmatic identification of potentially eligible clients, focusing on the presence of chronic conditions and perceived ADL task performance problems. Subsequently, formal eligibility was determined through systematic assessment against the prespecified inclusion and exclusion criteria by the study coordinator prior to baseline evaluation. Eligible clients were adults (≥ 18 years) diagnosed with one or more chronic conditions, living in own home, and able to interact relevantly and appropriately, communicate orally and in writing, and without severe cognitive deficits, aphasia, significant hearing loss, or dyslexia. To enhance retention, clients were required to confirm that they experienced decreased effort and/or spent extra time when performing ADL tasks, and express motivation to engage in OT [31] focused on adjusting everyday life to improve ADL task performance. Exclusion criteria included significant language barriers, known substance abuse, mental illness and/or acute conditions (< 3 months) dominating their ADL ability.

Occupational therapists delivering ADAPT were recruited from the rehabilitation centre, and required to have experience with practising according to the Occupational Therapy Intervention Process Model (OTIPM) [28], including addressing task performance problems by means of adaptational strategies [28, 35], be certified in administering the ADL-Interview (ADL-I) [36], calibrated Assessment of Motor and Process Skills (AMPS) raters [37, 38], and committed to adhere to the ADAPT manual.

Clients were recruited at the rehabilitation centre, through hospital referrals and municipality pathways, including general practitioners and home care staff. Municipal referral procedures involve assessing and aligning referrals with appropriate internal services to meet client needs. A recruitment rate of four clients per week was targeted to enrol 16 clients over 4 weeks (from week −4 to −1). Occupational therapists at the rehabilitation centre were informed about the study and provided with checklists to support screening and recruitment. Clients meeting the initial screening criteria were contacted by the study coordinator who presented the overall aim, content and design of the study, while continuing the screening process. Clients were explicitly informed about the three evaluation timepoints, including the overall content and duration of each evaluation, to support the client’s feelings of being adequately informed. Interested clients received written trial information and were asked to provide written informed consent prior to baseline evaluation.

Following baseline evaluations, clients were randomised to ADAPT or UOT using a stratified block design based on AMPS ADL motor ability measures. The study coordinator informed clients of their group allocation and scheduled follow-up evaluations. ADAPT participants were informed about the timepoints for intervention sessions delivered, while UOT participants received standard municipal communication via e-Boks (Denmark’s national secure digital mailbox system).

### Interventions

ADAPT 3.0 (Table 2) is a manualised, group-based OT intervention aimed at improving ADL ability in individuals with chronic conditions. Ten weekly group sessions and two booster sessions at weeks 16 and 24 are delivered by one or two ADAPT-trained occupational therapists to groups of six to eight participants. ADAPT follows a structured, stepwise problem-solving process [28, 39], in which the occupational therapists teach and guide a group of clients to (1) identify and analyse task performance problems, (2) develop and implement adaptational strategies (e.g. altered planning and prioritising, use of assistive devices, energy conservation, and adjustments to physical and social environments), and (3) reflect on outcomes. This process is primarily grounded in the OTIPM and explicitly informed by the Transactional Model of Occupation (TMO) [28], which underpins the programme’s focus on dynamic interactions between the person, the task, and the environment. Principles from Powerful Practice [28] further inform the educational design and learning strategies, emphasising client-centred and occupation-focused reasoning. ADAPT 3.0 includes standardised ADL assessments to provide a thorough and systematic ADL evaluation and to establish a solid foundation for intervention planning [30]. Information derived from the ADL evaluation enables occupational therapists to individualise group-based intervention sessions, while retaining formal session structures and shared teaching–learning strategies that combine education, practice and adaptation of task performance, individual reflection, and peer exchange.

Each session is anchored in manualised, session-specific programme theories, emphasising core session specific components essential for activating intended mechanisms and outcomes, to increase fidelity of programme delivery. Examples of session specific components included ‘explaining the purpose of OT’ (session 1), ‘informing about ineffective skills limiting task performance within the group’ (session 2), ‘teaching clients how elements within the task, the environment, and the person influence task performance’ (session 5). ADAPT is informed by evidence [21–24, 27], including findings from the related OT intervention programme, ABLE [30, 31], which share core mechanisms and theoretical foundations.

In March 2023, a 3-day ADAPT 3.0 workshop was conducted, in which four local occupational therapists were introduced to the programme’s theoretical foundations and preliminary evidence supporting its feasibility, acceptability, and effectiveness. The workshop included introduction of the overall programme content, emphasising detailed information about delivery of session-specific components, in order to increase fidelity, as well as the content of each session.

**Table 2:**
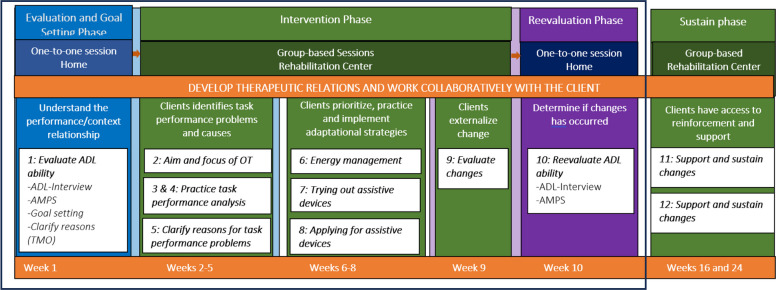
The 10-week ADAPT 3.0 program, and the two booster sessions

### Usual occupational therapy (UOT)

UOT was delivered flexibly as part of routine municipal practice, primarily in clients’ homes, but also in other local environments, at the rehabilitation centre, or by telephone.

Occupational therapists delivering UOT services were only informed about the pilot and feasibility study design and its overall focus. They were not assigned any research-related responsibilities, such as reminding clients about follow-up evaluation timepoints or supporting participants' progression through the trial. This approach was chosen to ensure that participants received an intervention consistent with municipal UOT services. However, one deviation from usual practice was that clients recruited for the study were not referred through the usual referral procedures. Consequently, no pre-specified intervention plans or goals were established prior to the intervention. Therefore, the occupational therapists were responsible for independently identifying clients’ task performance problems (ADL assessment), including needs evaluations, and formulating intervention goals.

### Study procedures

#### Data collection procedures

The occupational therapists delivering the ADAPT program were also responsible for conducting research-related activities to support data collection. During the first ADAPT session, occupational therapists informed clients about the scheduled follow-up evaluations at 12 and 26 weeks. Additionally, they were responsible for distributing and collecting registration forms at the end of each session.

#### Data collection

Data were collected to address the seven specific study objectives. Data collection methods, analyses, and progression criteria related to each aim are described in Table 1. Progression criteria were informed by previous studies [31], co-authors and municipal stakeholders [40, 41].

#### Recruitment and randomisation (objective #1)

Clients’ recruitment flow was documented as the number of clients screened and baseline evaluations conducted. Monitoring methods included registration of inclusion progress including reasons for declining or dropping out of trial participation. Lessons learned during the recruitment process were documented in logbooks.

To determine the acceptability of randomisation, a randomisation procedure was developed reflecting a procedure to be employed in a future randomised controlled trial. Following the baseline evaluation, each client’s AMPS ADL motor ability measure was recorded in a database designed to perform random stratification. The local study coordinator randomly assigned clients, using block sizes of 2 or 4, with a 1:1 allocation ratio, ensuring equal distribution of clients with low (< 1.0 logits) and high (> 1.0 logits) ADL motor ability measures across the two groups (ADAPT and UOT). The allocation sequence was concealed within the database, and group allocation could not be foreseen. The local secretary informed clients allocated to the UOT group according to standard municipal procedures by sending an electronic e-Boks invitation with the date of their first home-based UOT session.

Monitoring methods included registration of number of clients randomised and accepting group allocation, and randomisation progress, including reasons to refuse group allocation.

#### Trial participation (objective #2)

Clients were asked to rate the extent to which they felt adequately informed about (1) the purpose of the study, and (2) the number of evaluations and questionnaires embedded in trial participation. Responses were rated on a three-point Likert scale: 0 = inadequate, 1 = somewhat adequate and 2 = adequate, as part of the 12-week evaluation. Clients were encouraged to provide additional written information supporting their ratings.

#### Impact of trial on staff (objective #3)

The delivery of ADAPT was integrated into existing practice routines. Using registration forms, the ADAPT-trained occupational therapists reported any unanticipated consequences, such as negative side effects related to the delivery of specific ADAPT sessions. Responses were rated on a three-point Likert scale: 0 = no side effects, 1 = some side effects, and 2 = significant side effects. Occupational therapists were encouraged to provide written explanations of what occurred and how. Any negative side effects reported or observed during programme delivery were also documented in logbooks.

### Adaptation of trial conduct to local context (objectives #4 and #5)

To determine whether data on UOT could be extracted from client records, the local study coordinator extracted information using pre-specified variables related to dose, ADL evaluation, goal setting, and the content and focus of each UOT session. Also, the occupational therapists delivering ADAPT recorded if they experienced organisational or practical barriers to delivering each specific ADAPT session. Responses were rated on a three-point Likert scale: 0 = no, 1 = yes, of less importance, and 2 = of significant importance. Occupational therapists were encouraged to provide written information to elaborate on their responses. Information on UOT delivery and documentation, as well as barriers to programme delivery, was also documented in logbooks.

### Completion of measures (objective #6)

Primary, secondary [36, 37, 42–44], and exploratory [45] outcomes were evaluated at baseline and at 12 and 26 weeks from baseline. Baseline evaluations were conducted prior to group allocation, by the study coordinator, while follow-up evaluations were conducted by two external assessors employed at a research institute outside the rehabilitationcentre. 

*Primary outcome* was change in ADL motor ability, as measured by the AMPS [37] at week 12. *Secondary outcomes* included change in AMPS ADL motor ability (week 26) and changes in the following outcomes at 12 and 26 weeks: AMPS ADL process ability [37], ADL ability (performance and satisfaction), measured by the ADL-Interview (ADL-I, Performance and Satisfaction Scales) [36], Well-being, measured by the World Health Organisation-Five Well-Being Index** (**WHO-5) [46, 47], Health-related Quality of Life measured by the EuroQoL 5 dimensions (EQ-5D) [48, 49] and General Health, measured by the first question of the MOS 36-item Short Form health survey (SF-1 of SF-36) [43].

*Exploratory outcomes* were included to explore clients’ perceived transition and complement standardised outcomes. These outcomes were not included to provide confirmatory estimates of intervention effect. Baseline status for the domains covered by the exploratory outcomes was described using a Numeric Rating Scale (NRS). Exploratory outcomes were subsequently assessed using retrospective transition questions (TRANS-Q) at 12 and 26 weeks, where clients rated their perceived change relative to baseline on a 7-point Likert scale ranging from ‘markedly worse’ to ‘markedly better’. TRANS-Q explored clients’ perceived changes in ADL ability, problem-solving ability, assistance needed in everyday life, quality of life, and task equilibrium defined as the perceived balance between everyday task demands and the individual’s ability to manage ADL tasks, which represents a central target of adaptational OT interventions*.*

Completion rates for primary, secondary, and exploratory outcomes were recorded, along with the number of registration forms completed by clients and ADAPT-trained occupational therapists after each session. To evaluate data completeness of outcomes and registration forms, completion rates were calculated among clients who remained in the trial at the relevant assessment point. Reporting on completion of outcomes and registration forms from municipal stakeholders and outcome assessors was documented in logbooks.

To improve follow-up evaluation completion, clients were contacted the day before their scheduled appointment to confirm attendance. Questionnaires completed by the clients at baseline, 12, and 26 weeks were collected at the same time. To support completion of client registration forms for each ADAPT session, clients were given approximately 10 min at the end of each session to rate and comment on perceived mechanisms and outcomes. Likewise, occupational therapists were encouraged to complete their registration forms immediately after each ADAPT session.

### Fidelity and dose (objective #7)

Fidelity, defined as the extent to which ADAPT-trained occupational therapists delivered the programme as intended, was evaluated by registering the delivery of the manualised, session-specific key components considered essential to facilitate mechanisms of change and outcomes. Also, the occupational therapists recorded the number of ADAPT one-to-one and group-based sessions delivered (dose).

### Sample size

In line with established guidance for pilot and feasibility studies [50, 51], no formal sample size calculation was performed, as the study did not aim to estimate effect size or variability for the future Go:OT RCT. The feasibility objectives were process-focused and concerned trial conduct, i.e. recruitment, acceptability of randomisation, completion of measures, data extraction procedures, and intervention delivery. The statistical parameters required to power the definitive RCT, including assumptions for the AMPS ADL motor ability outcome, had already been determined from previously published data in a comparable population prior to this study [29, 37]. Feasibility data were therefore not intended to inform the RCT sample size.

The sample size was set pragmatically to meet the feasibility objectives and to allow piloting of trial procedures and intervention delivery under routine service conditions. One full ADAPT group (six to eight clients) was required. Allowing for an anticipated dropout rate of approximately 20%, the target sample was 16 participants (*n* = 8 ADAPT; *n* = 8 UOT), which was considered sufficient to address the predefined feasibility objectives.

### Data analysis

Descriptive statistics were employed to describe the study samples of clients and occupational therapists delivering ADAPT, and to address specific study aims (see Table 1 for details). Ordinal data will be reported in medians and range, whereas interval scale data will be reported in means and standard deviations (SD) given normal distribution.

## Results

### Participants

**Fig. 1:**
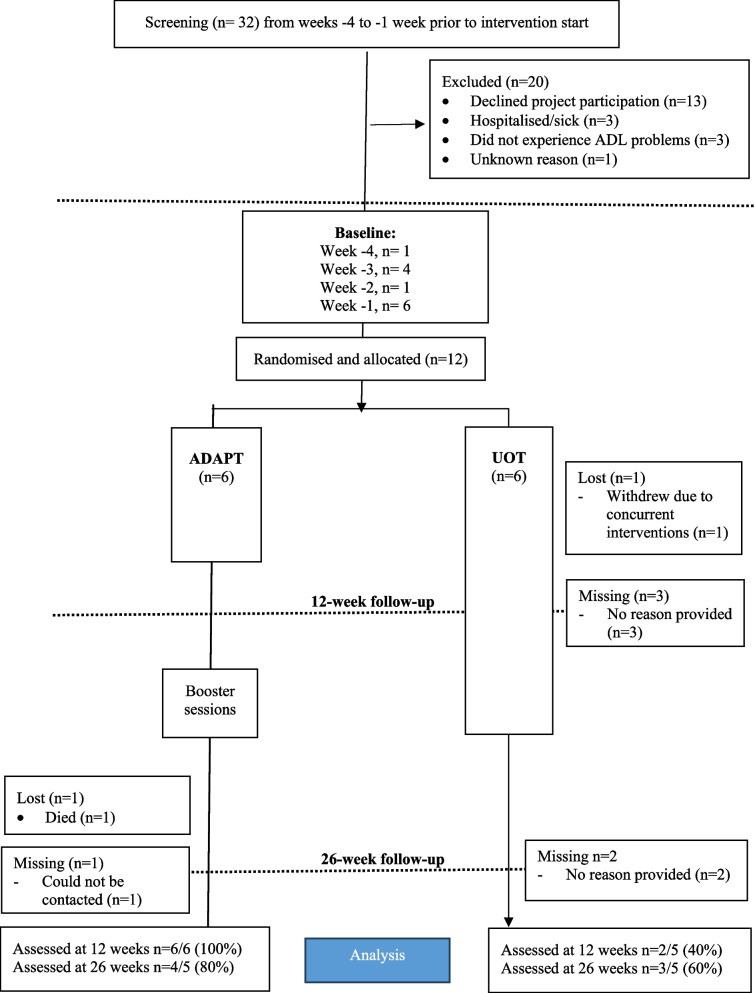
Consolidate standards of reporting trials (CONSORT) flow diagram of Go:OT trial design

This study included 12 clients (Fig. 1), four occupational therapists delivering ADAPT, and three occupational therapists delivering UOT. Clients were predominantly women, in their seventies, and primarily diagnosed with medical and musculoskeletal conditions, with a smaller proportion presenting neurological diagnoses (Table 3). Half of the sample had comorbidities (i.e. two or more conditions). Most participants were senior citizens or on early retirement and had a lower-level educational background. According to AMPS standardisation cut-offs (< 1.0 logits) [37, 52], mean ADL motor and process ability measures indicate increased physical effort, decreased efficiency, safety risk and a likely need for assistance in everyday life. The sample reported poor well-being (< 50) [44] and a fair general health (median 4) [43].

**Table 3:** Client (*n* = 12) characteristics when entering the study (baseline)

	**Total group** **(*n* = 12)**	**ADAPT group** **(*n* = 6)**	**UOT group** **(*n* = 6)**
**Gender:** Female, *n* (%)	10 (83)	5 (83)	5 (83)
**Age:** Years, median (range)	72 (50–94)	68 (50–92)	78 (52–94)
**Main diagnosis:** *n* (%)			
Medical	5 (42)	3 (50)	2 (33)
Neurologic	2 (17)	1 (17)	1 (17)
Musculoskeletal	5 (42)	2 (33)	3 (50)
Number of participants with comorbidity	6 (50)	2 (33)	4 (67)
**Civic status:** *n* (%)			
Living alone	7 (58)	4 (67)	3 (50)
Living with partner	5 (42)	2 (33)	3 (67)
**Job situation:** *n* (%)			
Senior citizen or early retirement	11 (92)	5 (83)	6 (100)
Employment assessment programme	1 (8)	1 (17)	0 (0)
**Educational level:** *n* (%)			
Lower-level education^a^	9 (75)	3 (50)	6 (100)
Higher-level education^b^	3 (25)	3 (50)	0 (0)
**Observed ADL ability (AMPS):** Logits, mean (SD)			
AMPS ADL motor ability	0.9 (0.8)	0.8 (0.2)	0.9 (0.4)
AMPS ADL process ability	0.9 (0.2)	0.9 (0.3)	0.9 (0.2)
**Self-reported ADL ability (ADL-I):** Logits, mean (SD)			
ADL-I, Performance	1.30 (0.83)	1.43 (0.64)	1.18 (1.06)
ADL-I, Satisfaction	1.19 (0.89)	1.49 (0.25)	0.90 (1.16)
**Well-being index (WHO-5):** Median (range)	44 (8–76)	52 (24–64)	40 (8–76)
**General health (SF-1 of SF-36**)**:** Median (range)	4 (3–4)	4 (3–4)	4 (3–4)

Medical: Lung disease (e.g. chronic obstructive pulmonary disease), cardiovascular disease, diabetes. Neurological: Stroke, multiple sclerosis. Musculoskeletal: Back/neck pain, shoulder pain, rheumatoid arthritis, osteoarthritis

^a^Collapse of three subgroups (primary school, vocational education, short higher education). ^b^Collapse of two subgroups (medium-term higher education, long higher education). *AMPS* Assessment of Motor and Process Skills, *ADL-I*, Activities of Daily Living Interview, *SF1 of SF-36* The first question in the MOS 36-item Short Form Survey Instrument, *TRANS-Q* Transition questionnaires, *UOT* usual occupational therapy, *WHO-5* The World Health Organization-Five Well-Being Index

The occupational therapists delivering ADAPT included three women and one man, aged 33 to 43. All therapists were certified ADL-I and calibrated AMPS users; however, one occupational therapist had only recently completed AMPS calibration and lacked clinical experience. All reported previous experience with OTIPM-based practice, and two had experience with group-based interventions. Their employment duration in the municipal setting ranged from less than a year to 7 years. UOT was delivered by three female occupational therapists aged 46–65 years, with more than 10 years of employment. All were familiar with the OTIPM; all were ADL-I certified and two were AMPS calibrated.

### Recruitment and randomisation

A total of 32 potential participants were screened, of whom 20 (62.5%) declined participation, primarily because they wished to continue the physiotherapy intervention already in progress and felt unable to engage in multiple concurrent interventions. Twelve (37.5%) clients were randomised, allocating six clients to ADAPT, and six to UOT. All accepted their group allocation and entered the respective interventions. The intended intake of four clients per week was not achieved (see Fig. 1 flow chart). Recruitment progressed more slowly than anticipated, for example, only one client was enrolled in week-4 due to a lack of eligible contacts, and in week-2, the study coordinator’s vacation limited recruitment to a single client. Consequently, recruitment could not rely solely on therapist screenings, as the coordinator needed to dedicate additional time to identifying and screening potential participants. Despite recruitment challenges, the overall progression criterion for recruitment was met.

### Trial participation

All six clients in the ADAPT group (100%) reported feeling adequately informed about the purpose of the study. Regarding the number of evaluations and questionnaires, five (83.3%) ADAPT clients reported being adequately informed, while one client reported being only somewhat adequately informed. Of the two UOT clients evaluated at the 12-week follow-up, one (50%) reported being adequately informed about the purpose of the study, while the other reported being only somewhat adequately informed. The same responses were provided regarding information about the number of evaluations and questionnaires embedded in the study. No written elaborations were provided. Thus, progression criteria were met for the ADAPT group, but not for the UOT group.

#### Impact of trial on staff

During the delivery of the ADAPT program, occupational therapists reported three instances of significant negative side effects. However, written elaborations revealed that these were not actual side effects, for example, ‘the occupational therapist had forgotten the ADL-I form’. Logbook data indicated that one ADAPT-trained occupational therapist found the one-to-one sessions involving AMPS evaluations too demanding due to limited clinical experience. This was identified as a significant negative side effect that hindered the delivery of AMPS in the one-to-one ADAPT sessions.

### Adaptation to local context

The occupational therapists registered eight organisational or practical factors as significantly hindering the delivery of the ADAPT program. However, only three of these were genuine organisational or practical issues. In session 3, the ADAPT session had to be relocated, because the usual room was reserved by colleagues. After session 7, an occupational therapist registered the need for access to a wider range of assistive aids, and in session 9, computer problems led to inefficient use of time. A significant implementation challenge concerned delivery of the one-to-one ADAPT sessions (sessions 1 and 10). According to the ADAPT manual, each one-to-one session was to be delivered within a 1-week time frame. In this pilot study, ADAPT was delivered to a group of six participants. As session 1 marked the start of the programme, all six session 1 visits were necessarily scheduled within the same week. The same applied to session 10 at the end of the programme, which likewise required all session 10 visits to be delivered within a single, separate week. This clustering of 2-h one-to-one sessions placed considerable demands on therapists’ time and scheduling flexibility.

Table 4 presents the type and amount of information accessible from clients receiving UOT. Data were accessible for more than 80% of the pre-specified variables across the six clients enrolled in the UOT group. Logbook registrations revealed that some occupational therapists reported difficulty in identifying clients’ ADL task performance problems, when intervention goals had not been established through the usual visitation process. Supporting these reports, the data extraction process revealed that only one client had been assessed using a standardised self-report instrument, none had been re-assessed using a standardised approach, and only three clients had clearly defined goals to inform their UOT intervention.

**Table 4:** Accessible information on usual occupational therapy (UOT), registered in client records *n* = 6 (100%)

Aspect	Pre specified information	Access to information
Dose	Number of contacts Type of contact (home/centre/phone) Duration of contact^a^ (minutes)	6 (100%) 6 (100%) 6 (100%)
Standardised ADL assessment	Applied methods^b^	6 (100%)
Goal setting	Goal identification	6 (100%)
Content of intervention	Intervention provided^c^	6 (100%)
Standardised ADL re-assessment	Applied methods^b^	6 (100%)
Adherence	Clinical judgement^d^	5 (83%)

^a^Includes registration of sessions being delivered in client’s home, at the rehabilitation centre or on telephone

^b^Includes self-reported/observation-based standardised ADL assessments

^c^Task performance, counselling and conversation about daily activity, conversation about other things than daily activity, training of body functions

^d^Occupational therapists having registered if (yes/no) the client has received adequately amount of occupational therapy

### Completion of measures

Table 5 presents data on the completion of baseline and follow-up evaluations among clients who remained in the trial at the relevant assessment points. All baseline evaluations were completed, meeting the progression criterion for that phase. Completion rates at 12- and 26-week follow-up evaluations were below the predefined progression criterion of 80% in the UOT group. Lower completion rates were observed due to missing outcome data, particularly in the UOT group, where 40% of clients were assessed at the 12-week follow-up. One client who did not attend the 12-week assessment participated in the 26-week follow-up, resulting in higher assessment rates at week 26 in the UOT group (60%). Logbook notes revealed that two clients from the UOT group contacted the rehabilitation centre to withdraw from the trial, stating that they did not find the UOT intervention meaningful or beneficial. Thus, the progression criterion was met for the ADAPT group, but not for the UOT group.

Although clients responded to the NRS and transition questions related to task equilibrium, many clients found this item difficult to comprehend. Consequently, the baseline and outcome assessors often had to assist clients in completing this item.

**Table 5:** Shows the evaluations applied in the Go:OT pilot and feasibility trial, as well as the numbers and percentage of evaluations conducted at baseline, 12- and 26-weeks follow-up

	Baseline Total, *n* = 12	12-weeks follow-up* ADAPT, *n* = 6/UOT, *n* = 5	26-weeks follow-up** ADAPT, *n* = 5/UOT, *n* = 5
AMPS, *n*, (%)	12 (100)	6 (100)/2 (40)	4 (80)/3 (60)
ADL-I, *n*, (%)	12 (100)	6 (100)/2 (40)	4 (80)/3 (60)
WHO-5, *n*, (%)	12 (100)	6 (100)/2 (40)	4 (80)/3 (60)
SF-1 of SF-36, n, (%)	12 (100)	6 (100)/2 (40)	4 (80)/3 (60)
EQ-5D, *n*, (%)	12 (100)	6 (100)/2 (40)	4 (80)/3 (60)
ADL ability-NRS, *n*, (%)	12 (100)	NA	NA
Task equilibrium- NRS, *n*, (%)	12 (100)	NA	NA
Problem-solving ability- NRS, *n*, (%)	12 (100)	NA	NA
Assistance needed NRS, *n*, (%)	12 (100)	NA	NA
Quality of life- NRS, *n*, (%)	12 (100)	NA	NA
ADL ability-TRANS-Q, *n*, (%)	NA	6 (100)/2 (40)	4 (80)/3 (60)
Task equilibrium-TRANS-Q, *n*, (%)	NA	6 (100)/2 (40)	4 (80)/3 (60)
Problem-solving-TRANS-Q, *n*, (%)	NA	6 (100)/2 (40)	4 (80)/3 (60)
Assistance needed-TRANS-Q, *n*, (%)	NA	6 (100)/2 (40)	4 (80)/3 (60)
Quality of life-TRANS-Q, *n*, (%)	NA	6 (100)/2 (40)	4 (80)/3 (60)

*One client dropped out of UOT, leaving a total of 11 to be evaluated at 12-week follow-up

**One client allocated ADAPT died, leaving a total of 10 clients to be evaluated at 26-week follow-up

*ADL-I *Activities of Daily Living Interview, *AMPS* Assessment of Motor and Process Skills, *NA* not applicable, *NRS* 10-point numeric Rating Scale, *SF1 of SF-3*6 The first question in the MOS 36-item Short Form Survey Instrument, *TRANS-Q* Transition questionnaires, *WHO-5* The World Health Organization-Five Well-Being Index

Registration forms were only to be completed by clients in the ADAPT group. All (100%) clients who had attended group sessions filled out the sessions-specific registration form. In the one-to-one session 1, one client missed a single item, explaining that she did not know how to respond. Another client did not receive the one-to-one session 10, and consequently, no registration form was completed by either the client or the occupational therapist. Thus, the progression criterion for completion of registration forms was met.

### Fidelity and dose

ADAPT registration forms completed by the occupational therapists showed that all group-based session-specific components (*n* = 19) were delivered (100%). In the one-to-one session 1, four clients received all five session-specific components (100%), while one client received four (80%). Registration forms indicated that the occupational therapists did not fully deliver the goal-setting component, as the client had no goals. Consequently, the occupational therapist defined goals on behalf of the client. A total of five clients attended the one-on-one session 10 in which all (100%) session-specific components were delivered. One client cancelled session 10 due to illness, and therefore, no components were delivered to that client. Thus, the progression criteria for fidelity and dose were met.

## Discussion

This pilot study evaluated aspects of the Go:OT trial design, conduct, and processes, such as recruitment, trial participation, impact on staff, adaptation to the local context, and measure completion rates, as well as feasibility aspects of the content and delivery of the revised ADAPT 3.0 (fidelity and dose). Although progression criteria were met for most objectives, there remains a need to refine processes related to recruitment, the information provided to clients in the UOT group, the structure of ADAPT 3.0, and the questionnaire items used to evaluate clients’ experience of task equilibrium. The results support that the updated ADAPT 3.0 is feasible and that previously identified fidelity issues have been resolved.

### Recruitment and randomisation

Recruitment through staff involvement proved challenging, making it difficult to consistently recruit four clients per week. Additionally, a significant proportion of potential participants declined to engage in the OT interventions, often citing a preference for physiotherapy and concerns about managing multiple therapeutic inputs simultaneously. Similar recruitment difficulties were reported in two studies evaluating the feasibility [53] and subsequently piloting [31] the ABLE intervention—an individualised, home-based OT programme targeting decreased ADL ability in individuals with chronic conditions. To address this issue, measures were implemented whereby the principal investigator became directly involved in providing trial information and conducting final eligibility screenings. This approach contributed to higher inclusion rates in the subsequent ABLE RCT [29]. This highlights the importance of carefully designed recruitment strategies that effectively communicate the value of OT in rehabilitation and allow clients to postpone trial participation until after completing their current physiotherapy.

The findings highlight that one study coordinator appeared too vulnerable, suggesting a need for a more robust and distributed recruitment infrastructure with shared research responsibilities. In future RCTs, baseline assessments could be delegated to other occupational therapists, allowing the study coordinator to focus on screening, clinician support, randomisation, and participant flow. Introducing a pre-screening phase prior to the formal screening period may further enhance recruitment by enabling early contact and preliminary eligibility assessments, creating a pool of potential participants for the main screening.

Despite recruitment challenges, all enrolled clients accepted randomisation and group allocation without objection. This may reflect participants’ perceived personal benefit and intrinsic motivation to contribute to research, which has been shown to significantly increase trial participation [54]. Furthermore, Jenkins et al. [55] identified trust in healthcare professionals and satisfaction with the information provided as key facilitators for patient engagement in RCTs. These relational factors were likely present in the recruitment process and may have contributed to participants’ willingness to accept randomisation. Moreover, randomisation was conducted via computer-based methods, which may have increased acceptability by ensuring transparency and reducing perceived bias. Kerr et al. [56] found that participants are more likely to accept randomisation when it is clearly explained and perceived as fair. Together, these findings suggest that once initial recruitment barriers are overcome, participant engagement can be sustained through a combination of perceived benefit, trust in professionals, and procedural clarity.

### Trial participation: adequate information

Unlike the previous feasibility study [21], clients in the ADAPT group were successfully informed, but the progression criterion was not met for the UOT group, which likely may be due to differences in post-allocation communication. In the ADAPT group, clients received standardised information about programme content and follow-up evaluation timepoints, reinforced by ADAPT-trained occupational therapists. In contrast, UOT occupational therapists were not instructed to provide similar details. While provision of standardised information may slightly compromise the ‘usual care’ aspect of UOT, it aligns with CONSORT guidelines for pilot trials [34] and ethical principles ensuring informed participation and data quality [57].

### Impact of staff/negative side effects

Although three negative side effects were initially reported during ADAPT delivery, qualitative elaborations revealed these to be misunderstandings or practical slips rather than true adverse effects. This highlights the value of supplementing side effect classifications with qualitative data [58]. One occupational therapist found the AMPS too demanding due to limited clinical experience, which hindered delivery. This aligns with findings by Kottorp et al. [59] and Isenberg et al. [60], showing that clinician experience and confidence impact fidelity. Supported by De Dios Pérez et al. [61], these insights underscore the need for either experienced and confident raters or robust support systems in trials involving complex assessments like AMPS.

### Adaptation to local context: practical factors

Although problems such as computer malfunctions and double-booked rooms may seem minor, they illustrate how logistical disruptions can affect the flow and perceived quality of intervention delivery. Implementing routines for checking equipment and room availability could enhance delivery in both ADAPT and UOT contexts. Similarly, ensuring access to a sufficient range of assistive devices is essential and should be addressed before the main Go:OT RCT.

The inability to deliver all one-to-one ADAPT sessions within 1 week, as prescribed in the manual, highlights a key challenge in complex intervention research, i.e. balancing fidelity to core components that ensure meaningful and feasible programme implementation in practice. As Moore et al. [62] emphasise, adaptations should be intentional, transparent, and co-developed with stakeholders to ensure feasibility and theoretical and methodological coherence. Hence, time-related adaptations should be embedded in the ADAPT program and manual to support internal validity and consistent delivery in the main trial.

### Adaptation to local context: access to UOT data

Client records provided access to UOT data, suggesting feasibility for describing UOT interventions in the main Go:OT RCT. However, the absence of standardised ADL assessments and infrequent goal setting may reflect limited adherence to core OT process models [28, 63], potentially reducing UOT’s effectiveness and client-centredness. 

Research related to evaluating the ABLE intervention programme [29, 30] supports that OT interventions guided by the theoretical OTIPM, including the use of ADL-I and AMPS in the initial evaluation phases, provide a robust basis for the entire therapeutic and problem-solving process, and contribute to intervention effectiveness [30]. That is, the apparent lack of systematic processes including standardised assessments in UOT may compromise outcomes and position more structured and standardised OT programmes as a stronger comparator in RCTs.

### Completion of measures

Completion of outcome evaluations was notably low in the UOT group. Logbook notes indicated that two participants actively withdrew due to perceived lack of relevance or benefit of UOT. Still, since no formal reasons for missing data were recorded, it remains unclear whether this reflects dissatisfaction with the UOT intervention, retention challenges, or procedural errors. To enhance trial fidelity and data quality in a future main trial, structured procedures for recording reasons for non-completion should be implemented. This aligns with recommendations for feasibility studies to identify and address barriers to retention and data collection [32].

Furthermore, drawing on Sekhon et al.’s Theoretical Framework of Acceptability (TFA) [64], it may be beneficial to assess participants’ perceptions of the intervention more systematically. Such data could help distinguish between procedural issues and intervention-related factors, thereby informing both interpretation of missing data and potential refinements to the intervention itself.

Outcome assessors also reported that participants struggled to comprehend the exploratory item related to ‘task equilibrium’. This suggests limited face validity and raises concerns about the relevance and clarity of this measure for the target population. In line with O’Cathain et al.’s guidance on feasibility studies [32], outcome measures should be both meaningful and comprehensible to participants. Therefore, this item should either be refined to improve clarity or omitted from the final trial to avoid compromising data quality.

### Fidelity and dose

The ADAPT 3.0 program was delivered with high fidelity to its core components. All group-based sessions were completed as planned, and most one-to-one sessions adhered closely to the manual. Illness prevented one client from attending a session, and another required substantial support to engage in goal setting, affecting delivery of these components. While inclusion criteria required participants to be motivated to improve ADL performance, difficulty in formulating goals could be interpreted as lack of motivation for change and hence constitute a recruitment challenge. Research, however, shows that individuals may be motivated for change but still struggle to articulate goals due to cognitive, emotional, or communicative barriers [65, 66]. A realist evaluation of ABLE [30] further highlights that therapists themselves may lack goal-setting skills, particularly in municipal contexts where goals are often predefined in referrals. Crawford et al. and Siegert and Taylor also emphasise that goal setting is a complex skill requiring support, and that client motivation alone is not sufficient. In such cases, therapist-led goal formulation, when done collaboratively and with client consent, is consistent with principles of client-centred occupational therapy and is supported by both theoretical [28] and empirical literature [65, 66]. According to the updated MRC guidance, fidelity should be assessed in relation to core components rather than rigid adherence, as adaptations are often necessary to ensure contextual fit. Nevertheless, these observations underscore the importance of clinicians’ competencies in collaborative goal setting, and the need for strengthening training or support in this area will be relevant to consider prior to a future full-scale trial. Fidelity thus encompasses not only adherence, but also practitioner competence and responsiveness. These findings suggest that the main trial should include attention to whether occupational therapists feel adequately prepared to support goal setting. To guide consistent registration, it may also be helpful to clarify when such situations indicate that a core component of the intervention is not being delivered as intended.

### Strengths and limitations

A key strength of this external pilot and feasibility study is the comprehensive evaluation of both trial processes and intervention delivery, including fidelity, dose, and contextual fit. Conducted in a real-world municipal rehabilitation setting, the study enhances ecological validity and provides valuable insights into the practical implementation of ADAPT, as well as the conditions necessary for conducting the Go:OT RCT. The use of multiple data sources, such as standardised evaluations, logbooks, registration forms, and client records, strengthens the credibility of the findings and supports the robustness of the main trial.

Several limitations should be acknowledged. Recruitment challenges and reliance on a single study coordinator may have introduced selection bias and limited sample representativeness. However, as a larger study with similar inclusion criteria reported comparable population characteristics [31], it seems reasonable to assume that the pilot sample reflects the broader target population. The study was conducted over a relatively short time period and included a small sample size and a single ADAPT group due to pragmatic constraints related to timelines and available resources. Furthermore, dropout and missed follow-up evaluations reduced the amount of available data. While the data were sufficient to address the primary feasibility objectives, limited insights were gained regarding potential changes in intervention fidelity, therapist workload, and trial adherence over time. Randomisation was stratified solely by ADL motor ability. Although this pragmatic choice was necessary to enable timely formation of group-based interventions, it may have resulted in residual imbalance in other characteristics known to influence ADL outcomes, such as age or educational level. Findings related to UOT should be interpreted with caution. The small sample size and the context-dependent nature of UOT, shaped by local organisational structures and clinical practices limit the generalisability of the findings beyond the specific municipal setting. Finally, incomplete documentation of reasons for non-adherence restricted a more nuanced understanding of attrition and missing data.

## Conclusion

This pilot and feasibility study suggests that the revised ADAPT 3.0 program and the overall Go:OT RCT design are implementable in a real-world municipal rehabilitation setting.

While most progression criteria were met, the study identified important areas for refinement, including recruitment procedures, minor structural programme adjustments, improved information for clients in the UOT group, and revisions in outcome measures. The findings support the readiness for a larger-scale evaluation, provided that the identified limitations are addressed.

## Data Availability

The datasets supporting the conclusions of this study are available from the corresponding author on reasonable request.
